# The effects of service dogs for children with autism spectrum disorder and their caregivers: a cross-sectional study

**DOI:** 10.3389/fpsyt.2024.1355970

**Published:** 2024-02-23

**Authors:** Kerri E. Rodriguez, Mandy Rispoli, Bridgette L. Kelleher, Evan L. MacLean, Marguerite E. O’Haire

**Affiliations:** ^1^College of Veterinary Medicine, University of Arizona, Oro Valley, AZ, United States; ^2^School of Education and Human Development, University of Virginia, Charlottesville, VA, United States; ^3^Department of Psychological Sciences, Purdue University, West Lafayette, IN, United States

**Keywords:** service dog, assistance dog, autism spectrum disorder, animal-assisted intervention, caregivers

## Abstract

**Introduction:**

Service dogs are an increasingly popular complementary intervention for children with autism spectrum disorder. However, despite increasing demand, there remains a lack of empirical research on their potential benefits. The purpose of this study was to evaluate the effects of service dogs on children with autism and their caregivers.

**Methods:**

A total of *N* = 75 families of children with autism were recruited from a non-profit service dog provider in the US, including *n =* 39 families previously placed with a service dog and *n =* 36 families engaging in usual care while on the waitlist. Caregivers completed an online survey containing both self- and proxy-report standardized measures of child, caregiver, and family functioning. Linear regressions modeled the relationship between service dog presence and survey outcomes, controlling for relevant child and caregiver covariates.

**Results:**

Results indicated that having a service dog was associated with significantly better child sleep behaviors, including better sleep initiation and duration and less sleep anxiety/co-sleeping with medium effect sizes. However, service dog presence was not significantly related to child withdrawal, negative emotionality, emotional self-control, hyperactivity, irritability, and lethargy with small effect sizes. For caregivers, having a service dog was not significantly related to standardized measures of caregiver strain, sleep disturbance, depression, or the impact of the child’s condition on family functioning with small effect sizes. Supplemental matched case-control analyses confirmed these findings.

**Discussion:**

In conclusion, service dogs were found to positively impact sleep behaviors among children with autism, but may not uniformly relate to other areas of child and caregiver wellbeing. Prospective longitudinal designs, larger sample sizes able to detect small effects, and studies that measure sleep using objective methods are needed to build on these findings.

## 1 Introduction

Autism spectrum disorder (ASD; autism) is a developmental condition characterized by persistent impairments in social interaction, verbal and nonverbal communication, and restricted/repetitive behaviors (1). A majority of caregivers of children and adolescents with autism will engage in home- and school-based interventions specific to the individual’s needs (2). In addition to evidence-based interventions to improve social skills and/or behavior, a majority of families also report engaging in complementary interventions (3, 4). One example of a complementary intervention for autism is animal-assisted intervention (AAI), a goal-oriented intervention that intentionally includes animals for therapeutic purposes (5).

Research evaluating the efficacy of AAI for children and adolescents with autism suggests that interactions with animals (including but not limited to dogs, horses, or small domestic animals) can significantly improve social interaction and communication (6, 7). While the theorized mechanisms for why animals may improve social outcomes for individuals with autism vary, it is suggested that animals can act as an initial social catalyst, or social bridge, to encourage communication with others (8–10). Research has also found that participation in AAI can result in increases in positive emotion, reductions in physiological stress, and reductions in aggressive behavior (7, 11). In this sense, animals may provide a calming presence, help maintain positive attentional focus, and reduce negative arousal (12–14).

In addition to AAIs, an increasingly popular practice in the autism community is the placement of a service dog (15). Service dogs are trained to perform tasks that directly assist an individual with a disability, including autism (16, 17). While service dogs may be self- or locally-trained, most placements occur by non-profit organizations that procure, train, and place service dogs for a specific disability (15). As of 2022, there are 64 non-profit organizations accredited by Assistance Dog International worldwide that place service dogs specifically for autism (18). These service dogs can be trained to interrupt self-stimulatory or repetitive behaviors, provide calming, deep pressure, and help ameliorate sensory overload. In addition to their trained tasks, service dogs may also benefit individuals with autism by increasing participation in daily activities (e.g., chores, caregiving actions, playing outdoors), assisting with the development and improvement of motor skills (e.g., throwing a ball, petting, and brushing), and facilitating social interactions with peers and the public (19–21).

Some research suggests that service dogs can provide psychological, social, and even physiological benefits for children and adolescents with autism, although findings have been mixed (22). In qualitative interviews, caregivers of children with autism describe that having a service dog has helped prevent or interrupt tantrums, improve sleep behaviors, prevent elopement behavior in public, and act as a calming and comforting presence (19–21, 23, 24). However, quantitative studies have reported mixed findings. A 2021 pilot study compared six families with a service dog to 12 families on a service dog waitlist and found no significant differences between groups on standardized parent-reported measures of child adaptive behavior or child social responsiveness (25). However, the sample size was small presenting challenges for analyses. A recent 2022 study assessing 11 families before and 2-3 months after placement with a service dog found improvements in parent-reported measures of child socioemotional behavior as well as decreases in parent and child physiological stress, but the study did not have a control group (26). In the largest study to date, a longitudinal study with 42 children with autism found that service dog presence was associated with lower cortisol levels and fewer problematic behaviors (27). In summary, although qualitative reports are promising, quantitative studies have produced mixed findings indicating a need for more research in this area (22, 28). Not only are studies needed with larger sample sizes and comparison groups, but it remains unknown how individual differences and circumstances may influence variability in findings (e.g., the child’s relationship with the service dog, time with the service dog, or the child’s social and communication behaviors).

While families often seek service dogs to benefit a child or adolescent with autism, some research indicates that service dogs can simultaneously benefit the lives of caregivers. Caregivers often serve as the primary handler for autism service dogs, creating a unique triadic relationship between the dog, child, and caregiver. Qualitative studies suggest that service dogs can improve caregivers’ quality of life by decreasing stress and providing them with a sense of safety and security (20, 21, 29). In qualitative interviews, caregivers also report that having the service dog in public increases the frequency and duration of family outings and can reduce isolation (19, 24).

However, similar to the literature on child outcomes, quantitative studies on caregiver effects have yielded mixed findings. One recent pilot study found evidence of reduced parenting stress for 11 caregivers of children with autism after 2-3 months with a service dog, but the study did not have a comparison group (26). In a large longitudinal study, 49 caregivers reported less parenting stress after nine months with a service dog compared to 49 caregivers remaining on the waitlist, however, the waitlist group had significantly higher parenting stress at baseline which confounded results (30). In contrast, a 2014 cross-sectional study found no difference in caregiving burden or strain among 134 caregivers of children with autism with a service dog compared to 87 on the waitlist (31). Due to the inconsistencies in findings and the limited number of studies conducted, there remains a need for more research on the effects of service dogs for caregivers and families of children with autism that integrates standardized measures, comparison groups, and large sample sizes. In addition, similar to research on child effects, it is unknown how the caregiver’s relationship with the service dog, time with the service dog, or the perceived costs of caring for the service dog may relate to variability in outcomes.

The present study aims to contribute to this literature base to characterize the effects of service dogs for children and adolescents with autism as well as their caregivers and families. The study’s aims were to explore the relationship between having a service dog on standardized measures of psychosocial functioning for individuals with autism (Aim 1) and their caregivers (Aim 2). We hypothesized that compared to those on the waitlist to receive a service dog, families with a service dog in the home would exhibit superior functioning in measured domains. In addition, an exploratory aim (Aim 3) examined how time cohabiting with the service dog, the child-service dog bond, the caregiver-service dog bond, and the perceived costs of the service dog may relate to child and caregiver outcomes.

## 2 Materials and methods

All protocols were reviewed and approved by the Purdue University Institutional Review Board (IRB Protocol #1906022320). As no interactions with the research team and service dogs occurred, a waiver was obtained from the Purdue University Institutional Animal Care and Use Committee.

### 2.1 Participants

Participating families were recruited from October 2019 to April 2021 from the database of service dog provider Canine Companions. Canine Companions is a 501(c) (3) non-profit organization accredited by Assistance Dogs International (ADI) which provides service and assistance dogs, including those for autism, free of cost to families across the US. Service dogs placed for autism are trained for various tasks, including retrieving, carrying, and delivering dropped items, responding to periods of self-stimulatory behavior, providing calming deep pressure, and performing interactive commands to promote social engagement with the child. Canine Companions service dogs are purpose-bred Labrador retrievers, Golden retrievers, or Labrador-Golden retriever crosses that follow ADI standards regarding canine health, temperament, and behavior. Canine Companions closely monitors and evaluates the health and welfare of service dogs both pre- and post-placement.

All child and caregiver participants recruited from Canine Companions had already been screened, interviewed, and approved to receive a service dog from the organization. Inclusion criteria to receive a service dog from Canine Companions includes caregiver age of at least 18 years old, child age of at least five years old, and a child diagnosis of an intellectual or developmental disability from a medical, psychological, or educational professional, which was self-reported by the caregiver. Inclusion criteria to be eligible to participate in the research study included child age of 5-18 years old and documentation of an autism diagnosis, including DSM-5 diagnoses of autism spectrum disorder as well as previous DSM iterations of Autistic disorder, Asperger syndrome, pervasive developmental disorder (PDD), or pervasive developmental disorder not otherwise specified (PDD-NOS).

The sample included families already placed with a service dog for a minimum of six months prior to recruitment in the study (*service dog group; n* = 39*)* and those on the waitlist to receive a service dog (*comparison group; n =* 36). Both groups received unrestricted access to usual care. Among the service dog group, time since placement ranged from 0.52 – 7.39 years (*M* = 3.68, *SD* = 1.99). The decision to exclude families with more recent placements (<6 months) was to ensure that any initial adjustment period had passed (25, 32). Time spent on the waitlist was not collected, but the average waiting time for the organization is roughly 1-2 years. Demographics for the sample of *N* = 75 families are displayed in **Table 1**, which were obtained via caregiver-report. Children were predominantly male (72%), with an average age of 11.25 years and range of 5-17. Average Social Communication Questionnaire (SCQ; see section 2.3.1) scores were 18.06 (*SD* = 5.89) across the total sample, with *n* = 63 of the 69 valid SCQ scores over the suggested cutoff of 10 for a likely autism screening (33). A majority of children had an associated condition in addition to an autism diagnosis, including limited verbal ability (75%), developmental delay (60%), learning disability (49%) and attention deficits (49%). The most common treatment services engaged in were speech and language therapy (61%), occupational therapy (48%), applied behavior analysis (43%), social skills training (20%), and parent-implemented interventions (20%). A subset of children took medications, including stimulants (28%), antidepressants (28%), antipsychotics (12%), anticonvulsants (15%), and antianxiety medications (15%). Children were mostly engaging in special education (45%) followed by general education (20%), part-time general/special education (19%), or home education (16%).

**Table 1:** Demographic characteristics of N = 75 participating families.

	Group				Group difference	
	Service Dog (*n* = 39)	Comparison (*n* = 36)	Total (*N* = 75)		*t* or X^2^	*p*
Child Demographics						
Age, *M (SD)*	12.92 (2.89)	9.44 (3.12)	11.25 (3.46)		5.014	<0.001
Male gender, *n* (%)	25 (64%)	29 (81%)	54 (72%)		2.514	0.113
SCQ, *M (SD)*	17.67 (5.68)	18.48 (6.17)	18.06 (5.89)		-0.573	0.568
Hours of education services per week, *M (SD)*	19.33 (13.19)	19.74 (11.66)	19.53 (12.39)		-0.408	0.684
Hours of treatment services per week*, M (SD)*	16.96 (31.39)	17.25 (36.87)	17.10 (33.86)		0.032	0.974
Caregiver Demographics						
Age, *M (SD)*	45.36 (5.41)	43.03 (6.18)	44.24 (5.87)		1.742	0.086
Female gender, *n* (%)	35 (90%)	34 (94%)	69 (92%)		0.562	0.453
# Children in home, *M (SD)*	1.85 (1.04)	1.97 (1.61)	1.91 (1.34)		-0.406	0.686
Pet dog in home, *n* (%)	13 (33%)	7 (19%)	20 (27%)		1.847	0.174
Race, *n* (%)					2.190	0.701
White	33 (85%)	26 (72%)	59 (79%)			
More than one race	3 (8%)	4 (11%)	7 (9%)			
Asian	1 (3%)	3 (8%)	4 (5%)			
Black or African American	1 (3%)	2 (6%)	3 (4%)			
American Indian/Alaskan Native	–	–	–			
Native Hawaiian or Pacific Islander	–	–	–			
Prefer not to say	1 (3%)	1 (3%)	2 (3%)			
Ethnicity, *n* (%)					1.131	0.568
Not Hispanic or Latino	34 (87%)	31 (86%)	65 (87%)			
Hispanic or Latino	4 (10%)	5 (14%)	9 (12%)			
Prefer not to say	1 (3%)	–	1 (1%)			
Education, *n* (%)					3.911	0.562
Post-graduate degree	20 (51%)	21 (58%)	41 (55%)			
4-year college degree	10 (26%)	10 (28%)	20 (27%)			
2-year college degree	5 (13%)	1 (3%)	6 (8%)			
Some college	2 (5%)	2 (6%)	4 (5%)			
High school/GED	1 (3%)	2 (6%)	3 (4%)			
Some high school	1 (3%)	–	1 (1%)			
Prefer not to say	–	–	–			
Annual Household Income, n (%)					3.943	0.684
Less than $25,000	1 (3%)	1 (3%)	2 (3%)			
$25,000 - $49,999	3 (8%)	1 (3%)	4 (5%)			
$50,000 - $74,999	4 (10%)	1 (3%)	5 (7%)			
$75,000 - $99,999	7 (18%)	8 (22%)	15 (20%)			
$100,000 - $124,999	4 (10%)	6 (17%)	10 (13%)			
$125,000 or above	17 (44%)	14 (39%)	31 (41%)			
Prefer not to say	3 (8%)	5 (14%)	8 (11%)			
Relationship Status, *n* (%)					8.955	0.062
Married	34 (87%)	32 (89%)	66 (88%)			
Divorced	5 (13%)	–	5 (7%)			
Widowed	–	2 (6%)	2 (3%)			
Single (never married)	–	1 (3%)	1 (1%)			
Living with significant other	–	–	–			
Separated	–	–	–			
Prefer not to say	–	1 (3%)	1 (1%)			
Employment, *n* (%)					3.187	0.562
Full-time	17 (44%)	19 (53%)	36 (48%)			
Part-time	11 (28%)	10 (28%)	21 (28%)			
Unemployed	9 (23%)	5 (14%)	14 (19%)			
Disabled or Retired	1 (3%)	1 (3%)	2 (3%)			
Prefer not to say	1 (3%)	1 (3%)	2 (3%)			

M, mean; SD, standard deviation; n, partial sample size; N, total sample size.

### 2.2 Procedures

A Canine Companions staff member contacted eligible caregivers from both the service dog group and the comparison group to ask for consent to share their information with the research team. The research team then directly communicated with participants to share study information and obtain verbal consent (caregivers) and assent (for children older than 12) to participate in the study. Caregiver participation consisted of completing an online survey via Qualtrics and collecting saliva samples from their child on three mornings (data reported separately). Caregivers were assured that neither their participation nor responses in the study would be shared with Canine Companions to ensure unbiased reporting. Participants were compensated $40 for survey completion. The recruitment rate was 84% (81 families consented to participate from 97 contacted), and the survey participation rate was 93% (75 families completed survey from 81 consented).

### 2.3 Survey measures

Demographic information collected for caregiver participants included age, gender identity, caregiving role, race/ethnicity, family size, presence of a pet dog in the home, employment, relationship status, annual household income, and level of education. Demographic information collected for child participants included child age, gender identity, associated conditions, participation in school or day programs, current treatments, and current medications taken.

#### 2.3.1 Child measures

The lifetime version of the Social Communication Questionnaire (SCQ; Chandler et al., 2007) was used to describe autism symptomology. The SCQ is a 40-item proxy-report questionnaire appropriate for both verbal and non-verbal children four years of age and older (34). Each item asks caregivers to report if their child experiences or exhibits a certain behavior with dichotomous response options (0, “No”; 1, “Yes”). For children with caregiver-reported language ability, summed scores range from 0 to 39, with a higher score indicating more severe autism symptoms. For children without language ability, summed scores range from 0 to 33. Scores of >15 indicate a potential identification on the autism spectrum. The SCQ had acceptable internal reliability (Cronbach’s α = 0.78). SCQ scores were not calculated for those with any missing data on the measure (occurring for *n* = 6 participants). No exclusions were made based on SCQ scores, as the SCQ is a screening measure rather than a diagnostic measure (35) and demonstrates reduced sensitivity and specificity among children and adolescents with co-occurring mental and behavioral diagnoses (33).

The Children’s Sleep Habit Questionnaire (CSHQ; 36) modified for children with autism (37) measured child sleep habits and behaviors. The modified 23-item scale has four subscales: sleep initiation and duration (SID), sleep anxiety/co-sleeping (SACS), night waking/parasomnias (NWP), and daytime alertness (DA). Caregivers were asked to indicate how often their child engaged in a range of sleep-related behaviors in the past week or typical week on a 5-point Likert scale, with a higher score indicative of worse sleep habits and behaviors. The modified CSHQ had good internal reliability (α = 0.85).

Behavioral and emotional difficulties were operationalized with two measures. First, the Aberrant Behavior Checklist (ABC; 38) measured children’s disruptive behaviors with the subscales of irritability, social withdrawal, and hyperactivity/noncompliance (47 items total). Questions asked caregivers to indicate the severity of child behaviors over the past four weeks on a 4-point Likert scale, with a higher score indicative of greater severity. The ABC had excellent internal reliability (α = 0.92). Second, The Behavior Assessment Scale for Children 3^rd^ edition (BASC-3; 39) measured child emotional behavior with the subscales of negative emotionality, withdrawal, and emotional self-control (24 items total). Questions asked caregivers to indicate the frequency with which the child has displayed behaviors “in the past several months” on a 4-point Likert scale, with a higher score indicative of higher frequency. The BASC had good internal reliability (α = 0.85). A BASC score was not calculated for *n* = 1 participant due to missing data.

The child’s quality of peer relationships was measured via the PROMIS^®^ (Patient-Reported Outcomes Measurement Information System; 40) Peer Relationships Pediatric Parent-Proxy Short Form (7-A v2.0). This 7-item measure asked caregivers to indicate the frequency with which their child engaged in social behaviors in the past week with peers on a 5-point Likert scale with higher scores indicating higher quality and quantity of peer relationships. This measure has been previously validated as an efficient and valid measure of peer relationships among youth with ASD (41). Scores were transformed to normative t-scores according to the PROMIS scoring manual with a population mean of 50 and standard deviation of 10. This measure had excellent internal reliability (α = 0.93).

#### 2.3.2 Caregiver measures

The Caregiver Strain Questionnaire (CGSQ; 42) measured caregiver strain. This 21-item measure has three subscales: Objective strain (OS), subjective externalized strain (SES), and subjective internalized strain (SIS). The three subscale scores are added to create a global score. Caregivers were asked about strain for themselves and/or their family in the past six months “as a result of their child’s emotional or behavioral problems” on a 5-point Likert scale, with higher scores indicative of higher caregiver strain. The CGSQ had excellent internal reliability (α = 0.93).

The PROMIS^®^ Sleep Disturbance Short Form 6-A (43) measured caregiver self-reported perceptions of sleep quality, sleep depth, and restoration associated with sleep. Caregivers reported on their sleep in the past week on a 5-point Likert scale, with higher scores indicative of worse sleep disturbance. Items were summed and transformed to normative t-scores according to the PROMIS scoring manual with a population mean of 50 and standard deviation of 10. The measure had good internal reliability (α = 0.89).

The Patient Health Questionnaire-9 (PHQ-9; 44) measured caregiver depression. This 10-item measure asked caregivers to indicate if they had been bothered by nine problems over the past two weeks on a 4-point Likert scale, with higher score indicative of more depression symptoms. The final item asked about the perceived difficulty of these problems interfering with daily life. The PHQ-9 had excellent reliability (α = 0.90).

The PedsQL™ (Pediatric Quality of Life Inventory) Family Impact Module Family Functioning Scale (45) measured caregiver-reported family functioning. The scale has two subscales: Daily Activities and Family Relationships. Caregivers were asked to indicate how often their family has faced a range of concerns and difficulties due to their child’s health in the past month on a 5-point Likert scale. Items were reverse-scored and linearly transformed to a 0–100 scale such that higher scores indicated better family functioning and less negative impact. This measure had excellent internal reliability (α = 0.90).

#### 2.3.3 Human-animal bond measures

Human-animal bond measures were given to the service dog group only. The Monash Dog-Owner Relationship Scale (MDORS; 46) perceived costs (PC) subscale measured the caregiver’s perceived costs of having a service dog. This 9-item subscale asked caregivers to indicate how inconvenient they perceived caring for and living with the service dog to be. Questions were scored on a 5-point Likert scale and summed such that higher scores indicated more perceived costs. The MDORS PC subscale had excellent internal reliability (α = 0.92).

The human-animal bond was operationalized with two scales intended to measure perceived closeness with the service dog in two distinct ways. First, the Monash Dog-Owner Relationship Scale (MDORS; 46) emotional closeness (EC) 10-item subscale measured the emotional closeness between the child and service dog as well as between the caregiver and service dog. The MDORS has been used in several studies of service dog-handler dyads (e.g., 47, 48) A higher score indicated higher child-service dog or caregiver-service dog emotional closeness. The MDORS EC subscale had good internal reliability (caregiver α = 0.87; child α = 0.91).

Second, the Inclusion of Other in Self (IOS) scale (49) measured the perceived interpersonal closeness, or interconnectedness, between the child/caregiver and service dog. The IOS is a single-item pictorial scale containing seven pairs of increasingly overlapping circles ranging from touching but not overlapping (1) to completely overlapping (7). The IOS has been used as a measure of the human-animal bond with both pet dog-owner dyads (e.g., 50) and service dog-handler dyads (e.g., 51). The IOS was chosen to complement the MDORS EC scale as it captures a purely subjective sense of closeness consistent with theoretical orientations of relationship psychology (52). In contrast, the MDORS EC subscale asks more objective questions to measure closeness such as frequency of specific behaviors and actions. Although the IOS and MDORS EC were significantly correlated (caregiver-dog: *ρ =* 0.517 *p* = < 0.001; child-dog *ρ =* 0.808, *p* < 0.001), measures were independently analyzed due to their unique conceptualization of closeness with the service dog.

### 2.4 Statistical analyses

All analyses were conducted in IBM SPSS version 28.0. First, demographic characteristics among children and caregivers in the service dog and comparison groups were compared using independent *t*-tests for continuous variables and chi-squared tests for categorical variables. Group-level statistics indicated that most demographic variables were not statistically different across groups. However, the service dog group was significantly older than the waitlist comparison group (M = 12.92 v. M = 9.44, *t* = 5.014, *p* < 0.001), had less prevalence of co-occurring developmental delay (49% vs. 72%, X^2 =^ 4.309, *p* = 0.038), and had higher use of antidepressants (39% vs 17*%*, X^2 =^ 4.141, *p =* 0.036). Therefore, these three variables were considered as covariates in statistical models.

Survey measures were examined for normality, and logarithmic transformations were performed for eight variables with a skewness statistic greater than twice its standard error (CSHQ Total, CSHQ SID, CSHQ SACS, CSHQ DA, CSHQ NWP, ABC Lethargy, PHQ, and CGSQ_SES). Multiple linear regression models were used to assess the association between service dog presence with child and caregiver measures. Survey measures were treated as dependent variables, while independent variables consisted of having a service dog or not (0, no; 1, yes) and child/caregiver covariates. Covariate inclusion was based on theoretical relevance to psychosocial outcomes and demographic variables in which significant group differences were found. For child models, covariates considered included gender identity (0, male; 1, female), age (continuous), presence of a developmental delay (0, no; 1, yes), and antidepressant use (0, no; 1, yes). For caregiver models, covariates considered included gender (0, female; 1, male), the number of children in the home (continuous), relationship status (0, not married or prefer not to say; 1, married), annual household income category (ordinal), and child autism severity as measured via the SCQ (continuous). To maximize power and ensure model parsimony, only covariates that were significant predictors at *p* < 0.10 were retained.

All models were checked for homoscedasticity, multicollinearity, and normality of residuals. A *post-hoc* power analysis conducted using G*Power (53) confirmed that the sample size (*N =* 75) was sufficient to achieve power of 0.91 to detect a medium effect (f^2 =^ 0.15) of the tested predictor (service dog presence) at an error probability of α = .05 with four predictors, which was the maximum number of predictors included across all models. We reported partial R^2^ as a measure of effect size, which describes the residual variance in each child or caregiver outcome explained by the service dog predictor. Partial R^2^ effect sizes were interpreted as small (< 0.02), medium (0.03 to 0.13), and large (> 0.14).

In consideration of bias due to the imbalance of confounding variables across the service dog and comparison group, supplemental analyses were conducted with a subset of matched participants. Matched groups of *n* = 24 in each group were created with SPSS case-control matching function on child age (tolerance of 2 years) and child gender (exact). Independent *t*-tests for continuous variables and chi-squared tests for categorical variables confirmed that demographic characteristics were equal across matched groups. Independent *t*-tests were then used to describe the association between service dog presence with child and caregiver measures.

Lastly, an exploratory aim assessed the relationship between service dog-related variables and child and caregiver outcomes among the *service dog* group only. Pearson’s bivariate correlations were conducted with child/caregiver measures and time since service dog placement, child-service dog emotional closeness (MDORS EC), caregiver-service dog emotional closeness (MDORS EC), and perceived costs of caring for the service dog (MDORS PC), which were all continuous interval variables. Nonparametric Spearman’s correlations were conducted with child/caregiver measures and child-dog and caregiver-dog interconnectedness (IOS), which was an ordinal variable.

## 3 Results

**Table 2** contains descriptive statistics for all child and caregiver survey measures. After controlling for covariates, there was a significant effect of having a service dog on child sleep habits and sleep behavior. Specifically, having a service dog was associated with significantly lower CSHQ scores (indicating better sleep outcomes; *p* = 0.038, medium effect size) including significantly better sleep initiation and duration (*p* = 0.005, medium effect size) and sleep anxiety/co-sleeping (*p* = 0.026, medium effect size). There was no significant effect of having a service dog on the CSHQ subscales of night waking/parasomnias or daytime alertness (*p*s > 0.380, small effect sizes). Service dog presence was not significantly related to child hyperactivity, irritability, and lethargy as assessed via the ABC (*p*s > 0.234), child emotional self-control, withdrawal, and negative emotionality as assessed via the BASC (*p*s > 0.184), or quality of the child’s peer relationships (*p* = 0.209; all small effect sizes). For caregivers, there was no significant relationship between service dog presence and total caregiving strain via the CGSQ, nor any of its three subscales (OS, *p =* 0.558, SES, *p* = 0.563, SIS, *p =* 0.416). There was also no significant relationship between service dog presence and caregiver sleep disturbance or depression symptoms (*p*s > 0.506) nor familial impacts due to the child’s health in terms of the family’s daily activities or family relationships (*p*s > 0.472; all small effect sizes).

**Table 2:** Descriptive statistics and linear regression results of child and caregiver measures.

	Service Dog (*n* = 39)	Comparison (*n* = 36)	Service Dog Effect^a^		
Child Measures	*M (SD)*	*M (SD)*	*B*	*Partial R^2^*	*p*
ABC Hyperactivity	18.03 (9.76)	21.56 (10.13)	0.048	0.002	0.696
ABC Irritability	13.46 (7.49)	13.64 (7.53)	0.157	0.018	0.234
ABC Lethargy	11.56 (8.98)	11.86 (7.82)	0.029	0.001	0.800
BASC Emotional Self-Control	12.69 (5.61)	12.64 (5.58)	0.088	0.006	0.506
BASC Withdrawal	14.26 (6.34)	14.14 (6.09)	-0.174	0.022	0.184
BASC Negative Emotionality	6.41 (3.73)	6.53 (3.03)	0.013	0.000	0.922
PROMIS Peer Relationships	31.79 (8.21)	31.56 (8.20)	0.169	0.021	0.209
Children’s Sleep Habit Questionnaire	33.26 (6.46)	37.00 (8.24)	-0.242	0.058	0.038*
Sleep Initiation and Duration	8.36 (2.37)	9.47 (2.51)	-0.377	0.106	0.005**
Sleep Anxiety/Co-Sleeping	5.82 (1.64)	8.31 (3.43)	-0.269	0.053	0.026*
Night Waking/Parasomnias	9.51 (2.81)	10.03 (2.93)	-0.090	0.008	0.444
Daytime Alertness	9.56 (3.19)	9.31 (2.92)	-0.117	0.010	0.380
Caregiver Measures	*M (SD)*	*M (SD)*	*B*	*Partial R^2^*	*p*
Caregiver Strain Questionnaire	7.80 (2.04)	8.05 (2.35)	-0.056	0.003	0.631
PROMIS Sleep Disturbance	52.47 (8.50)	53.74 (7.79)	-0.078	0.006	0.506
Patient Health Questionnaire	5.23 (4.97)	5.42 (4.90)	-0.066	0.004	0.564
PedsQL Family Impact - Daily Activities	41.24 (17.72)	42.59 (28.09)	-0.038	0.001	0.741
PedsQL Family Impact - Family Relationships	65.64 (21.28)	61.81 (27.49)	0.091	0.008	0.442

M, mean; SD, standard deviation; B, standardized regression coefficient; ABC, Aberrant Behavior Checklist; BASC, Behavior Assessment Scale for Children; PROMIS, Patient-Reported. Outcomes Measurement Information System; PedsQL, Pediatric Quality of Life Inventory; *, p < 0.05; **, p < 0.01.

a Reference category: waitlist (assistance dog=1; waitlist=0).

Supplemental analyses with the matched sample (*n* = 24 per group) showed identical findings to the above. Specifically, independent *t*-tests showed a significant association with service dog presence and total CSHQ scores (*t*(45) = -2.366, *p* = 0.011), CSHQ sleep initiation and duration (*t*(46) = -2.526, *p* = 0.008), and CSHQ sleep anxiety/co-sleeping (*t*(45) = -2.421, *p* = 0.011). However, there were no significant associations with service dog presence and all other child and caregiver survey measures (*p*s > 0.059). Full data from supplementary analyses are available upon request from authors.

### 3.1 Service dog group exploratory analyses

Exploratory analyses evaluated the relationship between time cohabiting with the service dog, the child-service dog bond, the caregiver-service dog bond, and the perceived costs of the service dog with child and caregiver outcomes (**Table 3**). The first variable examined was the time since service dog placement. There were no significant correlations between the time since the service dog was placed and any of the child (*p*s > 0.089) or caregiver (*p*s > 0.165) survey measures. Time since service dog placement was negatively correlated with child-dog emotional closeness (*p* = 0.010), child-dog interconnectedness (*p* = 0.006), and caregiver-dog emotional closeness (*p* = 0.014) such that newer service dog placements were associated with stronger child-dog and caregiver-dog bonds.

**Table 3:** Bivariate correlations between child and caregiver measures and service dog-related variables among *n* = 39 families with an assistance dog.

	Child-Dog Bond			
Child Measures	Time Since Placement	MDORS EC	IOS	
Social Communication Questionnaire	─	-0.391 *	─	
ABC Hyperactivity	─	─	─	
ABC Irritability	─	-0.366 *	-0.380 *	
ABC Lethargy	─	─	─	
BASC Emotional Self-Control	─	─	─	
BASC Withdrawal	─	─	─	
BASC Negative Emotionality	─	─	─	
PROMIS Peer Relationships	─	0.362 *	─	
Children’s Sleep Habit Questionnaire	─	0.377 *	─	
Sleep Initiation and Duration	─	─	─	
Sleep Anxiety/Co-Sleeping	─	0.341 *	─	
Night Waking/Parasomnias	─	0.415 **	─	
Daytime Alertness	─	─	─	
MDORS Child-Dog Emotional Closeness	-0.415 **			
IOS Child-Dog Interconnectedness	-0.430 **	0.810 ***		
	Caregiver-Dog Bond			
Caregiver Measures	Time Since Placement	MDORS EC	IOS	Perceived Costs
Caregiver Strain Questionnaire	─	─	─	0.332 *
PROMIS Sleep Disturbance	─	─	─	─
Patient Health Questionnaire	─	─	-0.349 *	─
PedsQL Family Impact - Daily Activities	─	-0.391 *	─	─
PedsQL Family Impact - Family Relationships	─	-0.379 *	─	─
MDORS Caregiver-Dog Emotional Closeness	-0.391 *			
IOS Caregiver-Dog Interconnectedness	─	0.524 ***		
MDORS Caregiver-Dog Perceived Costs	─	─	─	

ABC, Aberrant Behavior Checklist; BASC, Behavior Assessment Scale for Children; PROMIS, Patient-Reported Outcomes Measurement Information System; PedsQL, Pediatric Quality of Life Inventory; MDORS EC, Monash Dog-Owner Relationship Scale Emotional Closeness subscale; IOS, Inclusion of Other in Self Scale; ─, Not significant; *, p < 0.05; **, p < 0.01; ***, p < 0.001

Grey boxes indicate where a variable is being correlated with itself, thus no value is shown.

The second exploratory variable examined was the strength of the child-dog bond via emotional closeness (MDORS EC; *M* = 39.92/50, *SD* = 7.96) and interconnectedness (IOS; *M* = 4.97/7, *SD* = 1.71). Children with lower SCQ scores (indicating better communication skills and social functioning) were rated as more emotionally close with their service dog (*p* = 0.018) than children with higher SCQ scores. Children with less severe irritability on the ABC were rated as more emotionally close with their service dog (*p* = 0.024) and had higher interconnectedness with their service dog (*p* = 0.017). Children with a higher quality of peer relationships were rated as more emotionally close with their service dog (*p* = 0.024). Finally, children rated as more emotionally close with their service dog had worse sleep habits and behaviors (*p* = 0.020), including more sleep anxiety and co-sleeping behavior (*p* = 0.036) and more night waking and parasomnias (*p* = 0.010).

The last variable examined was the strength of the caregiver-dog bond via emotional closeness. (MDORS EC; *M* = 36.46, *SD* = 6.59), interconnectedness (IOS; *M* = 4.41, *SD* = 1.41), and perceived costs of caring for the service dog (MDORS PC; *M* = 12.77, *SD* = 5.67). Caregiver-dog and child-dog emotional closeness were significantly correlated (*p* = 0.016), but interconnectedness was not (*p* = 0.460). Caregivers with higher emotional closeness to the service dog reported more negative impacts of the child’s health on daily activities (*p* = 0.014) and family relationships (*p* = 0.017). Caregivers with higher interconnectedness with the service dog reported fewer depressive symptoms (*p* = 0.030). Finally, caregivers that reported more perceived costs of caring for the service dog reported higher caregiver strain (*p* = 0.042).

## 4 Discussion

This study aimed to evaluate the effects of service dogs on children with autism and their caregivers. Using a cross-sectional design, we compared families of children with autism with a service dog to families on the waitlist, both of which were receiving usual care. After controlling for child and caregiver covariates, having a service dog was significantly associated with better child sleep behaviors, including better sleep initiation and duration and less sleep anxiety/co-sleeping behaviors with medium effect sizes observed. However, service dog presence was not significantly associated with child social and emotional behaviors, child peer relationships, caregiving strain, caregiver sleep, and family functioning, with small effect sizes observed. We discuss these results in depth below.

### 4.1 Child findings

Results of the current study found that living with a service dog was associated with significantly better parent-reported sleep initiation and duration and less sleep anxiety/co-sleeping among children with autism. These findings align with qualitative studies describing how children are more likely to sleep through the night due to a service dog’s presence in their room or bed (24, 26) and are willing to stay in their room by themselves (20). Our results support the hypothesis that service dogs provide a sense of security and comfort to a child with autism at night, which may translate into exhibiting less sleep anxiety and co-sleeping behavior with a caregiver. Curiously, although the service dog group reported better sleep outcomes on average, within-group analyses suggest that children who were more emotionally close to their service dogs had worse sleep outcomes. However, this finding is correlational, not causational; it may be that children who are struggling with sleep seek out more of a connection or develop a closer bond with their service dogs. Future research will benefit from examining sleep more closely in this population, including assessing the service dog’s effect on sleep quality, awakenings, and duration using objective methods, as well as examining the role of the child-service dog bond on outcomes in longitudinal designs.

Contrary to our hypothesis, having a service dog had no significant association with child and adolescent social withdrawal, irritability, or hyperactivity behaviors via the ABC or negative emotionality, withdrawal, and emotional self-control behaviors via the BASC. Notably, the sample size was not powered to detect small effects, and the heterogeneity of the child sample likely contributed to high variability that may have also obscured a small effect. While some qualitative studies have described improvements in child and adolescent social and emotional behavior after being placed with an autism service dog (20, 21, 23, 24), these benefits may be too variable across individuals to capture in a group comparison design, especially considering the large range in child behavior due to the spectrum nature of autism. In addition, socioemotional behaviors among children with autism have subtle variations in quality and frequency, making it hard to reliably measure change (54). Future research will benefit from considering a clustering approach to explore how specific autism phenotypes may respond differently to a service dog intervention (55, 56), integrating longitudinal, within-person designs, and larger sample sizes to detect small effects.

Contrary to our hypothesis, there was no significant relationship between having a service dog and the quality of children/adolescents’ peer relationships via the PROMIS Peer Relationships scale. However, it is notable that both service dog and waitlist groups had scores that were almost two standard deviations below the population mean of 50 (mean t-scores of 31.56 and 31.79, respectively), indicating the quality and quantity of peer relationships to be low in the current sample. Due to the limitations in caregiver report, it is possible that this measure did not capture the subtle social facilitation effects that may occur while children are at school or away from home (41). Given the social facilitation effects that service dogs (57, 58) and therapy dogs (59) have been reported to provide to children with autism, future research will benefit from complementing caregiver-report scales with teacher-report scales and observational measures (e.g., 10) or incorporating other types of social facilitation measures.

For families with a service dog, indicators of the child-dog bond were high and similar to other service dog populations (e.g., 60). Interestingly, correlational analyses found newer service dog placements were associated with stronger child-dog and caregiver-dog bonds. This may be due to a novelty effect such that excitement and initial engagement with the service dog could lead to higher perceptions of closeness, which may stabilize over time. Qualitative studies have described how some children form immediate strong bonds with their service dogs while others may take more time due to physical or social constraints (19, 61). Future, longitudinal research will be valuable to examine how the child-dog bond forms and changes over time. Correlational analyses also found that children with lower SCQ scores (indicating higher social functioning) had a stronger emotional bond with their service dog. It may be that children/adolescents with more verbal and nonverbal communication skills tend to interact with or talk to their service dog more, leading to higher caregiver perceptions of the child-dog bond. Indeed, research suggests that there are differences in how individuals with autism interact with animals depending on their social abilities and preferences. For example, an observational study of 16 children with autism interacting with a service dog for the first time found evidence of different subgroups: those that preferred more tactile contact with the service dog, those that preferred more vocal contact with the service dog, and those that relied on parental direction (62). Future research is needed to explore the relationship between autism phenotypes (e.g., eye contact preferences, sensory profiles, and social skills) and the development and maintenance of the child-service dog bond.

### 4.2 Caregiver outcomes

The second aim of this study was to assess the association of having a service dog on caregiver and family wellbeing. Contrary to our hypotheses, having a service dog in the home was not associated with caregiver-reported objective or subjective strain as a result of their child’s emotional or behavioral problems, with small effects observed. Although qualitative studies have described how caregivers experience less stress from the sense of security provided by a service dog (20, 21, 24, 57), quantitative findings have been mixed. In fact, our findings mirror that of a larger cross-sectional study that compared families with and without an autism service dog on Caregiver Strain Questionnaire (CGSQ) scores (31). This discrepancy between qualitative and quantitative findings may be due to the nuances of caregiving burden. For example, qualitative data from the current study (57) as well as from other studies (19, 31, 58) suggest that service dogs can alleviate perceived stress for caregivers, but may also exacerbate or maintain current levels of caregiving pressure due to added dog-related needs. Future research will benefit from examining the impacts of service dogs on other more nuanced caregiving constructs such as caregiver satisfaction, which has been found to be sensitive to service dog placement in other caregiving populations (e.g., 63).

Caregivers who reported more perceived costs of the service dog (including financial costs, increased responsibility, and restrictions placed on the caregiver because of the dog) also reported higher caregiver strain. This finding mirrors a recent survey study of over 600 parents of children with autism which found a significant correlation between parents’ perceived burden of having a pet and parents’ self-reported stress (64). Indeed, caregivers of children with autism experience higher caregiver strain compared to caregivers of children with other disabilities (65). Importantly, the cross-sectional design of this study precludes inferences about causal relationships between caregiver strain and perceived costs of caring for the service dog. It is unknown whether strained caregivers perceive the service dog as more burdensome, or if caregivers that find the service dog to be burdensome develop more caregiving strain. Of note, the sample of caregivers in the current study were mostly White, educated, married, and of middle to high socioeconomic status. Given the evidence surrounding racial and ethnic disparities in autism (66), a more diverse sample is needed to gain a comprehensive understanding of the caregiving experience as it relates to service dogs. Future longitudinal research is also necessary to determine the role that financial, personal, and emotional costs of caring for a service dog may play in exacerbating or relieving caregiver strain, parental stress, and overall quality of life.

There was no significant effect of service dogs on caregiver sleep disturbance, including sleep quality, sleep depth, and restoration associated with sleep, with small effect sizes observed. This finding is particularly notable given that service dogs were associated with better *child* sleep behavior, including better sleep initiation and duration and less sleep anxiety/co-sleeping with the caregiver. Indeed, qualitative studies have found that caregivers of children with autism with a service dog in the home report improvements to their own sleep due to indirect effects of improvements in the child’s sleep (20, 24). It is possible that the brief self-report measure chosen for this study (PROMIS Sleep Disturbance) did not capture these carry-over effects, or that the effect was too small to detect statistically. However, it is also important to note that caregivers in both groups had average levels of sleep disturbance (52.47 and 53.74) compared to the population average of 50, indicating that sleep disturbance was not common in this population and thus may not have been sensitive to change following service dog placement. Future research will benefit from pursuing more complex measurements of caregiver sleep, including sleep anxiety, child co-sleeping behavior, and objective measures of sleep quality and quantity.

Contrary to our hypothesis, there was no significant relationship between service dog presence and the severity of the negative impacts of the child’s autism symptoms on the family’s daily activities or relationships, with small effect sizes observed. Although some qualitative studies have suggested improvements to family functioning from autism service dogs (20, 21, 24), these studies primarily describe benefits to familial relationships and stress independent of the child’s symptoms. Similarly, qualitative findings from the current study found that service dogs were described as a catalyst for improved family interactions by co-regulating with individual family members and providing a source of joy (57). Therefore, the measure chosen in the current study (PedsQL) may not have captured these nuanced impacts on family functioning, or the small sample size was not adequate to detect a small effect. Future studies may benefit from integrating more holistic family functioning scales that capture variability in the quality and quantity of familial interactions as well as longitudinal, larger-scale designs.

Interestingly, the higher the emotional closeness between a caregiver and the service dog, the more negative impacts the child’s condition had on family activities and relationships. This may be due to the possibility that caregivers experiencing familial difficulties may be more likely to turn to the service dog as a source of support. This finding aligns with a previous study on caregivers of individuals with a mobility or medical service dog in which worse caregiver-reported psychosocial health was associated with higher emotional closeness with the service dog (67). This pattern has also been observed in studies of pet dogs in which a stronger human-animal bond has been associated with more psychological distress (68) and lower levels of positive experience (69). Future research should more closely examine the role of the caregiver-service dog bond, including its development and maintenance, as well as its implications for family functioning and caregiver wellbeing.

### 4.3 Limitations & future directions

This was a cross-sectional, single time point study and groups were not systematically matched on all demographic characteristics; we are unable to establish any causal relationships between variables. Longitudinal, randomized designs will be required to determine the causal effect of service dogs on child and caregiver outcomes. Second, caregiver-reported outcomes may have been influenced by self-reporting biases such as social desirability or recall bias. However, we could not integrate child self-report measures due to child/adolescent age and verbal ability differences. Future research will benefit from using objective measures of sleep, physiological biomarkers, observational methods, and more “real-time” data collection such as ecological momentary assessment to measure how service dogs impact child, caregiver, and familial functioning beyond self-report survey measures (70). Similarly, future studies integrating individualized assessments and objective methodologies (e.g., physiological biomarkers, wearable technology) will be needed to both characterize individual dog welfare (71) and to examine how these variables may impact child and/or caregiver outcomes.

Other limitations of this study pertain to sample characteristics. First, although all efforts were made to maximize sample size, the target population was limited in number, and sample size was relatively low. Specifically, the sample size had inadequate power to detect any small effects. Therefore, it may be that the study was underpowered to describe the subtle effects of service dog-related change in the constructs measured. Second, we relied on documentation of an autism diagnosis for children instead of conducting a standardized diagnostic assessment such as the Autism Diagnosis Observation Schedule (ADOS), which would have provided more accuracy. Child participants in this study were also heterogenous regarding co-occurring conditions and medications and treatments received. However, this is common in research with this population (72) and also represents a more ecologically valid sample that is representative of those who are placed with a service dog. Notably, our sample was a self-selected convenience sample that had actively sought out and applied for a service dog. Therefore, it is unclear how these results may generalize to children and adolescents with autism that are *not* amenable to a service dog. The sample is also not representative of the larger population of families of children with autism; caregivers were mostly White-identifying, non-Hispanic, highly educated (81% with a Bachelor’s degree or higher) and of relatively high socioeconomic status (74% with an annual income of $75,000 or higher). Lastly, data collection for this study occurred during the coronavirus (COVID-19) pandemic, which may have influenced child, caregiver, and family outcomes.

### 4.4 Conclusion

In conclusion, this study adds to a limited but growing knowledge base on the effects of service dogs for children with autism and their caregivers. This exploratory cross-sectional study found that having a service dog was associated with better child sleep behaviors, suggesting that this should be a focus of increased research in this area. Specifically, research should further explore the effects of service dogs on child sleep quality, quantity, and disturbances using objective methods. We did not find significant associations between having a service dog and child social and emotional behavior, child peer relationships, caregiving burden, caregiver sleep, caregiver depressive symptoms, and family functioning, which were all observed with small effect sizes. It is possible that these null findings may reflect inherent challenges of naturalistic waitlist study designs or the application of standardized measurements to an individualized intervention in a heterogenous population with a small sample size. Larger prospective, randomized studies building on these initial findings will be necessary to fully evaluate the effects of service dogs on child and caregiver outcomes.

## Data availability statement

The raw data supporting the conclusions of this article will be made available by the authors, without undue reservation.

## Ethics statement

The studies involving humans were approved by Purdue University Institutional Review Board (IRB Protocol #1906022320). The studies were conducted in accordance with the local legislation and institutional requirements. Written informed consent for participation in this study was provided by the participants’ legal guardians/next of kin. The animal studies were approved by Purdue University Institutional Animal Care and Use Committee. The studies were conducted in accordance with the local legislation and institutional requirements. Written informed consent was not obtained from the owners for the participation of their animals in this study because no interactions occurred between service dogs and the research team.

## Author contributions

KR: Conceptualization, Funding acquisition, Investigation, Methodology, Writing – original draft, Writing – review & editing. MR: Conceptualization, Methodology, Writing – review & editing. BK: Conceptualization, Methodology, Writing – review & editing. EM: Conceptualization, Methodology, Writing – review & editing. MO: Conceptualization, Funding acquisition, Investigation, Methodology, Supervision, Writing – review & editing.
